# The Limits of Infants’ Early Word Learning

**DOI:** 10.1080/15475441.2019.1670184

**Published:** 2019-10-01

**Authors:** Loukia Taxitari, Katherine E. Twomey, Gert Westermann, Nivedita Mani

**Affiliations:** a Department of Secondary General Education, Ministry of Education and Culture, Nicosia, Cyprus; b Division of Human Communication, Development and Hearing, University of Manchester, Manchester, UK; c Department of Psychology, Lancaster University, Lancaster, UK; d Georg-Elias-Müller Institute for Psychology, University of Göttingen, Göttingen, Germany; e Leibniz ScienceCampus Primate Cognition, Göttingen, Germany

## Abstract

In this series of experiments, we tested the limits of young infants’ word learning and generalization abilities in light of recent findings reporting sophisticated word learning abilities in the first year of life. Ten-month-old infants were trained with two word-object pairs and tested with either the same or different members of the corresponding categories. In Experiment 1, infants showed successful learning of the word-object associations, when trained and tested with a single exemplar from each category. In Experiment 2, infants were presented with multiple within-category items during training but failed to learn the word-object associations. In Experiment 3, infants were presented with a single exemplar from each category during training, and failed to generalize words to a new category exemplar. However, when infants were trained with items from perceptually and conceptually distinct categories in Experiment 4, they showed weak evidence for generalization of words to novel members of the corresponding categories. It is suggested that word learning in the first year begins as the formation of simple associations between words and objects that become enriched as experience with objects, words and categories accumulates across development.

Infants begin to solve the puzzle of language learning from a very young age. Even before the onset of speech, in the second half of the first year, the young language learner is equipped with crucial phonetic/phonological abilities, such as fine-tuning to language-specific phonological contrasts (Werker & Tees, 1984), the ability to segment words in fluent speech contexts (Jusczyk & Aslin, 1995) and sensitivity to the prosodic and phonotactic characteristics of their native language (Mattys & Jusczyk, 2001). These fundamental abilities allow infants to take on the challenge of learning word-object associations well before the onset of full-fledged productive language skills (Gershkoff-Stowe & Smith, 2004; Golinkoff et al., 2000; Hollich, Hirsh-Pasek, Golinkoff, & Al, 2000; Sloutsky, 2009; Waxman, 2002; Waxman & Lidz, 2006; Werker, Cohen, Lloyd, Casasola, & Stager, 1998). Indeed, there is empirical support for some level of language understanding in pre-speech infants. For example, Tincoff and Jusczyk (1999) showed that upon hearing the words *mommy* and *daddy*, six-month-old infants oriented to a video of their mother or their father, respectively, but not to other women and men. In a subsequent study with the same age group, the authors showed that infants could link the words *hand* and *feet* to videos of adult hands and feet (Tincoff & Jusczyk, 2012). Bergelson and Swingley (2012) and Syrnyk and Meints (2017) have demonstrated word recognition for several common objects in infants at or before 9 months of age, documenting the formation of word-object associations through daily experience with language. Further, Twomey and Westermann (2018) demonstrated that 10-month-old infants could learn novel-label to novel-object mappings when trained with objects and labels over the course of a week.

These results show impressive word learning abilities of infants at the end of the first year: the ability to associate labels with objects, but also the ability to generalize familiar words to new, previously unseen objects from the same category. The ability to generalize, or extend a label to a novel category member, is critical to infants’ later language proficiency: infants must not only be able to recognize familiar referents but also to recognize unfamiliar members of familiar categories. While there is substantial evidence that young infants can quickly form categories (for a review, see Gershkoff-Stowe & Rakison, 2005), and object category formation is facilitated by the presence of labels (Fulkerson & Waxman, 2007), the specifics of the development of label generalization in the first year of life is less clear. On the one hand, it appears that very young infants require substantial experience with word-category mappings in order to do so. For example, Schafer (2005) showed that after extensive training over a three-month period from their caregivers at home, nine-month-old infants generalized words to novel exemplars of the trained category. On the other hand, studies using ERP to examine word learning suggest that even nine-month-olds may be able to generalize new labels to novel exemplars of the object category (Junge, Cutler, & Hagoort, 2012).

Against this background, the current study tests word learning and generalization from minimal exposure in the early stages of word learning at 10 months. In a series of hybrid looking time/3D object examining studies, we first tested whether infants were familiar with two word-object associations by presenting them with images of two objects side-by-side, accompanied by the label for each object in successive trials. Following this pre-test, we trained infants on these word-object pairings by presenting them with 3D objects accompanied by the appropriate label. Finally, we presented infants with a habituation task in which they encountered 2D images of objects accompanied by the appropriate label. At test, infants were presented with either the same two objects they had been trained on or two novel instances of the object category accompanied by the label.

The following sections describe four variations of the task which probe the limits of infants’ early word learning and generalization. Experiment 1 tested arguably the simplest word learning scenario by examining whether 10-month-old infants can learn word-object mappings from single object exemplars. Experiment 2 explored whether, like toddlers, infants’ word learning benefits from training with multiple exemplars. In Experiment 3, we asked whether infants could generalize a novel label to a new member of the learned category based on the same training that resulted in word learning in Experiment 1. Finally, in Experiment 4, we asked whether training infants with perceptually distinct objects boosted generalization. The results offer a fine-grained picture of the fragile beginnings of infant word learning and generalization.

## Experiment 1: can 10-month-olds learn two new word-object associations?

### Method

#### Participants

Twenty-nine infants (13 girls) with mean age of 10.04 months (range: 9.16– 10.90, *SD*: 0.35) were included in the final sample. Seven additional infants were tested but excluded due to fussiness. All participants were healthy, full-term infants, without any known visual or hearing problems, from monolingual English-speaking households. Infants’ parents were recruited at the maternity ward of the local hospital and contacted by telephone at the time of the study. Participants were given a t-shirt or bib as a gift for their participation.

#### Stimuli

For the purposes of our studies, we selected naturally occurring objects, in an effort to facilitate word learning in a laboratory setting and allow infants to exhibit generalization of newly-learned word-object associations, by reducing the cognitive load imposed by the task. We therefore selected the word-object pairings *cow* and *horse*. These items were chosen as they are likely to be familiar to infants in their first year of life, through picture books, toys, songs and television shows. This visual familiarity might facilitate learning of label-object associations in-task. At the same time, *cow* and *horse* were reported as unlikely to be understood by English-learning 10-month-olds: according to the Wordbank database, which includes data from the widely-used MacArthur-Bates Communicative Development Inventory (Fenson et al., 1994), *cow* and *horse* are understood by only 8% and 3% of 10-month-old American infants, respectively. Thus, here we attempt to benefit from visual familiarity with the objects but not from familiarity with the word-object associations. To ensure this, we presented infants with a pre-test to rule out the possibility that they knew these word-object associations prior to the experiment see *Procedure*). Images of these animals were perceptually similar (i.e., their colors and shapes were similar) and we did not therefore expect any systematic preferences in infants’ responses to the two stimulus types.

The specific stimuli for all experiments were chosen on the basis of adult visual similarity and typicality rating studies. Two groups of adults were presented with seven different images of cow toys and seven different images of horse toys and asked to rate how good an example of a word each instance was (*typicality rating study, N* = 20, 10 women) or how similar pairs of within and across category items were (*visual similarity rating study, N *= 20, 16 women). The original 3D toys were naturalistic animal figures made by the German toy producer *Schleich*. The cows measured 13cm long and 7cm tall and the horses 12cm long and 10cm tall. Test stimuli were digitized photographs of the toy animals in side view, presented on a white background on a computer monitor.

For the typicality rating study, the objects, their labels and a typicality scale were simultaneously presented on screen. Participants were asked to rate how good an instance of the category, to which the printed word belonged, the presented object was. This was done using a Likert-type scale of 1 (not good) to 7 (very good) by pressing a button from 1–7 on the computer keyboard. Objects, labels and the scale remained on the screen until participants had pressed a button. Of the seven cows and horses presented, participants rated two cows and two horses with noticeably lower typicality scores relative to the others (mean typicality scores less than 5). The remaining five cows and five horses were all rated above 5, ranging from 5.31 to 6.71 for cows and 5.44 to 6.73 for horses. These tokens were considered typical of the corresponding categories and chosen as the stimulus items for the experiments with analyses of visual similarity and subsequent infant experiments. For Experiment 1 in particular, we chose the cow-token (C1 – mean rating = 6.71) and the horse-token rated most typical (H4 – mean rating = 6.73; see Table A1, Appendix for a complete listing of the typicality ratings for cows and horses).

For the visual similarity rating study, participants were presented with pairs of within or across category objects along with a similarity scale from 1 (very dissimilar) to 7 (very similar). No labels were presented in this study. Participants were instructed to rate how similar the two pictures looked as quickly as possible by pressing a button from 1 to 7 on the computer keyboard. The pictures and similarity scale remained on the screen until participants had made a decision. They were told that even though pictures could belong to different categories, they could look more similar to pictures from the same category. For example, a golden retriever and a German Shepherd can look very similar, but both look very different to a European cat. However, both the retriever and the German Shepherd also look very different to a chihuahua, which in turn could look more similar to the European cat. We only used the category-typical items (based on the typicality ratings) in the visual similarity study since these were the only items to be presented to infants in the experiments. Each participant’s ratings of the similarity of each cow with each other cow were averaged. For each participant, this average was compared to the mean of his or her ratings when considering a cow and a horse. Across participants, the within-category ratings for *cow* averaged 5.1 (*SD* = 1.0) and the between category ratings 3.1 (*SD* = 0.8). This difference in ratings was significantly greater than zero (*t*(19) = 7.66, *p* < .001). A similar comparison considered within-category *horse* ratings (mean 5.2, *SD* = 1.1). These similarity ratings were also greater than the between-category ratings (*t*(19) = 7.56, *p* < .001). The specific objects chosen as the stimuli for Experiment 1 (C1, H4) were rated as being dissimilar to one another (mean rating = 2.9/7, *t*(19) = − 8.79; *p* < .001).

Audio files were recorded in a single session by a female speaker delivering them in infant-directed speech. The audio recordings were made with a digital audio tape recorder (DAT) in a soundproof recording booth. Audio stimuli were digitized at a sampling rate of 44.1 kHz and a resolution of 16 bits and spliced using Goldwave v. 5.10. All audio stimuli were presented at a typical 65 – 69dB as measured during stimulus presentation by a sound level meter located approximately where the infant’s head would be during the experiment. Labels were produced by the speaker both in citation form and embedded in sentences. In the training block, infants heard the words both in isolation and embedded in the following carrier phrases: *It’s an X!, Look at the X!* and *Do you remember the X?* In test blocks, labels were presented in isolation, in order to make the task easier for infants (Jusczyk, 1999).

Infants were presented with both pictures of objects and real objects. 3D objects were the original toy objects depicted in the preceding adult studies. Pictures were digital photographs of the objects on a white background. Photographs were digitally processed to remove any background noise using Adobe Photoshop CS2 and saved in 24-bit bitmap format. For training blocks, pictures were processed using Macromedia Flash 5 in order to add motion to the objects, which moved 45 pixels up and down the vertical axis in a smooth identical movement. The distance was covered over 25 frames, changing every 100 ms. In test blocks, pictures were stationary. All items were saved looking in two directions, by flipping the canvas horizontally for both still and moving images; this ensured that items always faced the center of the screen whether presented on the right or left side.

#### Procedure

The experiment was divided into a pre-test phase, interactive play-phase, an on-screen training phase and a test phase. Aside from the interactive play phase – which took place in a colorfully decorated room directly outside the testing booth – infants and parents were seated in a dimly lit booth. Infants sat on their parent’s lap approximately one meter away from a 110cm x 40cm screen. Pictures measured 30cm x 30cm and were presented on the left and right of the screen, separated by a 28cm gap. Audio stimuli were presented through two speakers located just above the center of the screen. Parents were asked not to interact with their child during the session. They were instructed to close their eyes and listened to music through headphones. Two cameras above each picture recorded infant eye movements, each sending a signal to a digital router to form a single twin split-image of the infant. A computer captured the whole session for later off-line scoring. The experimenter manually controlled the experiment, which proceeded at the individual infant’s own pace. A tone delivered through the speakers served to re-capture infants’ attention between trials.

##### Pre-test

Because it was likely that infants could have had some experience with exemplars of our stimulus categories, the pre-test was designed to establish whether they had learned the word-object association outside the lab. Infants were tested on their prior knowledge of the word-object associations using a standard inter-modal preferential looking task (e.g., Golinkoff, Hirsh-Pasek, Cauley & Gordon, 1987). Pre-test consisted of four trials in which one picture of each object was presented side-by-side on screen for five seconds (e.g., cow and horse). Exactly 1s into the trial, infants heard the word *Look!* followed by the target word at 2.5 s. Each picture was the target twice, that is, infants heard *cow* on two trials and *horse* on two trials, with order of presentation of trials randomized and side of presentation of the target image counterbalanced within participants.

##### Interactive play-phase

After the pre-test, infants were taken out of the booth for an interactive play phase, during which they were allowed to actively manipulate two 3D objects, one at a time, while hearing the label for this object. This procedure has been shown to enhance the word-learning process and help infants quickly learn new word-object pairings in the laboratory (Mani & Plunkett, 2008; Pruden, Hirsh-Pasek, Golinkoff, & Hennon, 2006). It was therefore included, in addition to the classic on-screen training phase which followed, in order to help these 10-month-olds quickly form new associations. During the interactive play-phase, parent and infant were seated in a colorfully decorated room and the experimenter sat opposite them at approximately 50 cm distance from the infant. The infant remained seated on the parent’s lap, and the parent was instructed not to interact with the infant at all. The interactive play-phase lasted approximately 4–5 minutes. During this period infants were allowed to actively manipulate the objects (one at a time), while each object was labeled six times using the following sentences:

Oh! Look! This one’s a X! Look! It’s a X! Do you want to play with the X? Do you like the X? Oh! Look! Where’s the X? Look! Here’s the X!

Each object was presented separately and the order of presentation was pseudo-randomized so that half of the infants saw the cow first and half saw the horse first.

##### On-screen training phase

**Figure 1.**
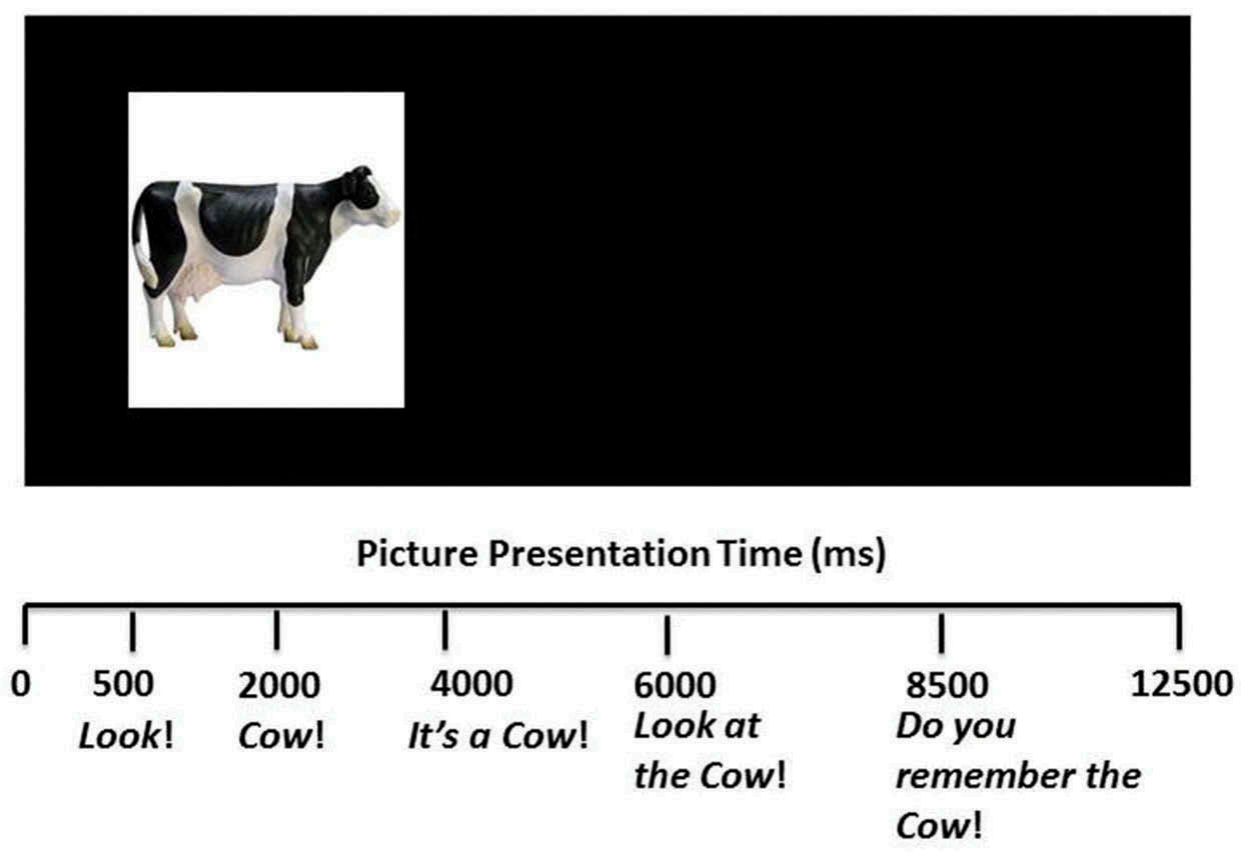
Example of an on-screen training trial. The picture remains on the screen for 12.5s, while the target word is presented four times.

After the interactive play-phase, infants were taken back into the booth for an on-screen training phase in which they were presented with up to 20 trials, divided into five sub-blocks of four trials. Each training trial presented infants with a single moving image of one of the objects on one side of the screen and the label for this object. Each trial lasted 12.5 s with the following phrases appearing at the following times: *Look!* (0.5 s); [*target word*] (2 s); *It’s a* [*target word*]*!* (4 s); *Look at the* [*target word*]*!* (6 s); *Do you remember the* [*target word*]*?* (8.5 s). This process was repeated for each item separately (see Figure 1).

Each sub-block of four trials consisted of two training trials for each word-object pairing. The order of presentation of the sub-blocks was randomized. Side of presentation of the pictures on-screen was counterbalanced within sub-blocks. Order of presentation was randomized within sub-blocks such that no more than four same-picture trials appeared consecutively, and no more than two consecutive same-picture/same-side trials appeared (for the effect of alternating picture side on maintaining infant interest in the task, see Canfield, Smith, & Brezsnyak, 1997). Pictures in the training block moved smoothly up and down the vertical axis, in order to increase their perceptual salience and facilitate word learning (Werker et al., 1998). Left-right looking was scored on-line by a trained researcher blind to the side of presentation of the target. The first sub-block was considered to be the baseline according to which all subsequent sub-blocks were compared. Training ended when looking time had fallen below a pre-defined criterion (decrease below 65% from the first sub-block of four trials) or when all twenty trials had been completed. On average, infants in this experiment needed 3.87 sub-blocks (range: 2–5, *SD*: 1.25) to reach the habituation criterion. The counterbalanced blocking of trials ensured equal exposure to both word-picture pairings before training stopped. A post-hoc analysis confirmed that infants who habituated had done so to both of the pictures, not just one of them. The criterion of 65% has been used in previous word learning studies, and is a good index of infants’ decreasing attention, while ensuring that they are still engaged in the task (Fennell & Werker, 2004). Fifteen infants reached the habituation criterion.

##### Test phase

Infants were finally tested in a post-training test block consisting of four trials, similar to the pre-test block. Here, infants were presented with two images of the objects side-by-side on-screen for five seconds. Exactly 1s into the trial, infants heard the word *Look!* followed by the target word at 2.5 s. Each picture was the target twice; that is, infants heard the label *cow* in two trials and the label *horse* in two trials, with order of presentation of trials randomized and side of presentation of the target image counterbalanced within participants.

#### Measures

A digital-video scoring system was used to assess videos of the infants on a frame-by-frame basis (every 40 ms). Infant looking behavior was scored off-line by a trained scorer; 25% of trials were scored again by the same scorer and another experienced scorer blind to the side of target presentation. Frame-by-frame scored trials across all four experiments reported here were entered into a Cohen’s kappa analysis revealing substantial interrater agreement, *k* = .80 (95% CI [0.78– 0.81]).

The coded video frames were used to determine the amount of time infants spent looking at the target (T) and distracter (D) images. As is standard in the infant literature, we calculated the proportion of time (T/(T + D)) infants spent looking at the target – a *Proportion of Target Looking* measure (PTL). Infants’ eye movements were analyzed separately for the pre- and post-naming phase. The post-naming phase began 360 ms following the onset of the label, in order to ensure that only those eye movements that could reasonably be considered a response to the auditory stimulus were included in the analysis (Swingley, 2009). To ensure equal pre- and post-naming phases, the pre-naming phase also began 360 ms into the trial. Both trial phases lasted until the end of the 2500 ms, leaving in the analysis 2140 ms pre- and post-naming. The *effect of naming* is the difference in the PTL from the pre- to the post-naming phase: a significant increase would suggest that infants correctly associate the target labels with their intended images. Means and standard deviations for the pre- and post-naming phases were calculated over trials and across participants. Table 1 presents the means and standard deviations for all experiments in the pre- and post-training Test Blocks, respectively. One infant was excluded from this analysis due to not providing any data in the post-naming phase of the pre-test block. Shapiro-Wilk tests confirmed that PTL did not depart from a normal distribution in both test phases (*p*s > .05).

**Table 1.** Means and standard deviations for all experiments in the post-training Test Block.

Experiment	Test Block	Test Phase	Mean (SD)
1	Pre-training	Pre	.52 (.13)
	Pre-training	Post	.45 (.25)
	Post-training	Pre	.48 (.11)
	Post-training	Post	.57 (.20)
2	Pre-training	Pre	.49 (.12)
	Pre-training	Post	.47 (.18)
	Post-training	Pre	.52 (.12)
	Post-training	Post	.55 (.19)
3	Pre-training	Pre	.49 (.15)
	Pre-training	Post	.49 (.12)
	Post-training	Pre	.49 (.11)
	Post-training	Post	.46 (.14)
4	Pre-training	Pre	.44 (.14)
	Pre-training	Post	.49 (.20)
	Post-training	Pre	.44 (.11)
	Post-training	Post	.50 (.17)

### Results

We submitted the proportion of target looking to a 2 (Trial Phase) x 2 (Test Block) x 2 (Habituation Group) repeated measures ANOVA with Trial Phase (pre- vs. post-naming) and Test Block (pre- vs. post-training) as within-subjects factors and Habituation group (Habituators (*N *= 15) vs. Non-Habituators (*N *= 14)) as a between-subjects factor. There were no main effects of Trial Phase, *F*(1,27) = 0.084, *p* = .77, and Test Block, *F*(1,27) = 1.31, *p* = .26, but there was a significant interaction between Trial Phase and Test Block, *F*(1,27) = 5.16, *p* = .03, *η^2^* = .16. There were no main effects of or interactions with Habituation group and any of the other factors (*p*s > .22). On the basis of the significant interaction, we analyzed data from the pre- and post-test blocks separately. Data were collapsed across Habituation Group in subsequent analyses.

**Figure 2.**
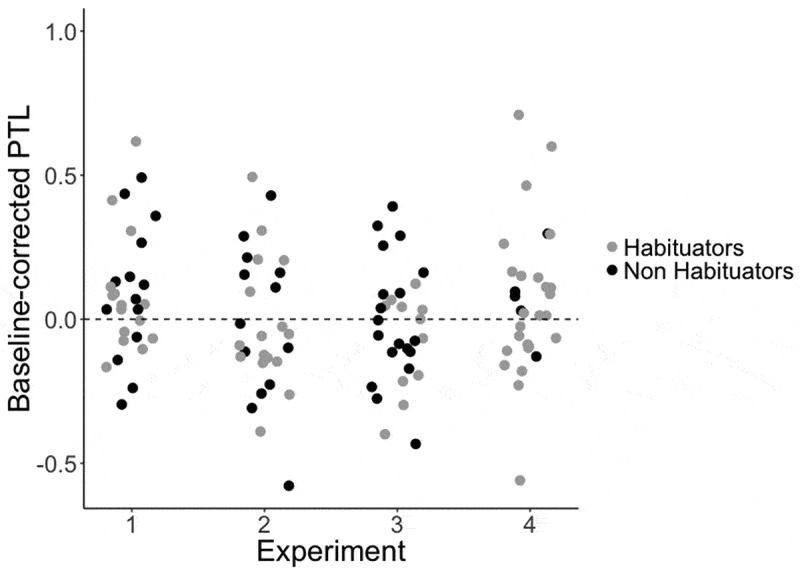
Target looking in Experiments 1–4: Difference in the proportion of target looking between the pre- and post-naming phases of the trial for test blocks in Experiments 1–4. Habituators and non-habituators are presented separately.

Post-hoc analyses using paired-samples two-tailed *t*-tests revealed no difference between the pre- and post-naming trial phases in the pre-test block, *t*(28) = 1.19, *p* = .24, 95% CI [−0.05, 0.18], offering no evidence in support of the claim that infants had prior knowledge of the to-be learned word-object associations. In contrast, a significant difference in looking times between the two trial phases was found in the test block, *t*(28) = − 2.24, *p* = .03, 95% CI [−0.17, −0.01], indicating that infants successfully formed the association between the object and the label (see Figure 2, *cow: M* (increase from pre- to post-naming phase) = .09, *SD *= .44, *horse: M *= .10, *SD *= .31).

**Figure 3.**
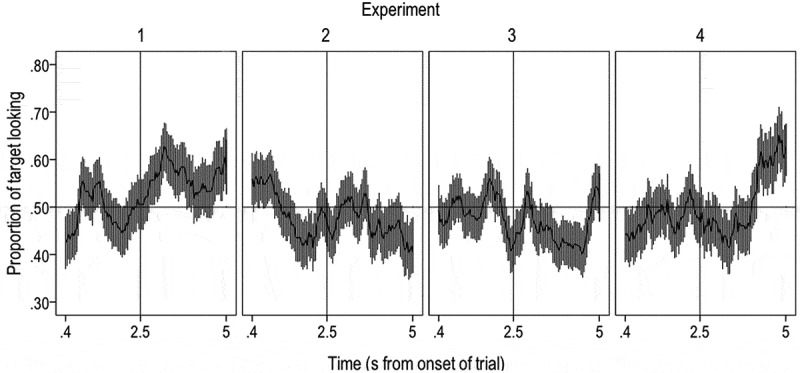
Timecourse plot showing the proportion of target looking (y-axis) during the whole trial in the post-training test phase in the four experiments. The time is presented in milliseconds (x-axis). The vertical line at 2.5s shows the onset of the presentation of the target word. The gray lines present the one standard error above and below the mean.

Figure 3 is a timecourse plot that shows the proportion of target looking before and after the presentation of the target word in the test block in milliseconds. The vertical line at 2.5 s indicates the onset of the presentation of the target word. This shows a consistent preference for the target picture (above 50%) in the post-naming phase of the trial, supporting the results of the ANOVA showing an increase in target looking after the presentation of the target word.

### Discussion

Experiment 1 set out to investigate whether 10-month-old infants could learn two word-object associations. We found that infants showed no evidence for comprehension of the word-object associations in the pre-test block. This result suggests that the infants had limited prior knowledge of the to-be-learned word-object associations or, if they did have some experience with the objects and words, their knowledge of the word-object associations was not strong enough to be evident at test. Following training, however, infants looked longer at the labeled object compared to the distracter object at test. In other words, infants at 10 months can quickly learn an association between an object and its label in a brief experimental session.

These findings extend previous research by testing the limits of young infants’ word learning capacities. A number of previous studies have shown that young infants can learn at least one novel word-object association in a laboratory setting (e.g. Pruden et al., 2006; Woodward, 1994), There are, however, crucial differences between these previous studies and the current study; in particular, it is difficult in such cases to examine whether infants have correctly associated the novel label with the object, or whether they are simply attending to the labeled object because this object is made more salient by having been given a label. Here, we show that 10-month-olds show systematic looking behavior – which is typically interpreted as word recognition – for both novel word-object associations in a brief experimental session. We suggest that this presents particularly strong evidence in favor of young infants’ early word learning proficiency.

Studies examining the neurophysiological correlates of infant word learning have tested infants’ learning of multiple word-object associations (Junge et al., 2012). These studies, however, did not test whether infants were familiar with the word-object associations beforehand. Since, like us, they tested whether infants can learn labels for objects that they may have seen before in their daily environment, it is possible that infants were already to some extent familiar with the to-be-learned word-object associations. Here, we first tested whether infants knew these mappings before training and found no evidence of infants’ prior knowledge of the associations. Thus, our finding that infants can learn the novel word-object associations directly results from the training that infants received during the experiment.

In Experiment 2, we extend this to examine how the input available to children modulates word learning, with particular regard to exposure to multiple exemplars from the same category.

## Experiment 2: can infants learn new label-object associations from multiple exemplars?

In a series of 3D object word learning tasks, Twomey, Ranson, and Horst (2014) trained 30-month-old toddlers with novel word-object mappings and demonstrated that children retained words only after encountering multiple perceptually similar exemplars of each novel object category. We reasoned that 10-month-old infants may likewise benefit from exposure to multiple category exemplars. On the other hand, it is possible that the additional cognitive demands imposed by processing multiple different referents may disrupt 10-month-old infants’ ability to learn word-object associations. Experiment 2, therefore, examines infants’ word learning given exposure to multiple exemplars of the object category during training.

### Method

#### Participants

30 infants participated in the study (15 girls) with mean age of 9.80 months (range: 9.16– 10.40, *SD*: 0.26). All participants were healthy, full-term infants, without any known visual or hearing problems, growing up in monolingual English-speaking households. Infants were recruited in the same way as infants in Experiment 1. Participants were given a t-shirt or bib as a gift for their participation.

#### Stimuli

The stimuli for Experiment 2 were identical to the stimuli for Experiment 1 aside from the on-screen training phase. Unlike in Experiment 1, infants in Experiment 2 were presented with multiple exemplars of the novel object categories during training. Training items were the four most typical items based on the ratings obtained from adult speakers of English (C1, C2, C3, C5 and H1, H3, H4, H5; see Appendix). All items were rated above 5 on the Likert scale. Visual similarity ratings of the different cow and horse tokens presented during training in Experiment 2 are presented in Table A2–A4, Appendix. As confirmed by the analysis reported in Experiment 1, adult participants found the different cow and horse tokens to be more similar to tokens from the same category than tokens from different categories. Note that during training, infants were also presented with the items that would be presented at test since the focus of this experiment was not to test infants’ generalization of a newly learned label to a novel exemplar, but rather to examine whether young infants can learn a novel word-object association from exposure to multiple exemplars of the object category. Thus, infants in Experiment 2 saw four different typical cows and horses during training, one of which was also presented at test (the same token presented at test in Experiment 1).

#### Procedure

The procedure was identical to Experiment 1 except for the online training phase. The on-screen training block differed from Experiment 1 in that four exemplars from each category were used, instead of a single item. Within each sub-block of the training block, infants were presented with one cow and one horse exemplar. The four cows and four horses in the training block were paired with each other exhaustively across infants and order of presentation of the sub-blocks varied randomly within-participants, in order to avoid similarity effects. Eleven infants habituated in this experiment. On average infants in this experiment needed 4.60 sub-blocks (range: 3– 5, *SD*: 0.62) to reach the habituation criterion.

#### Measures

Infants’ left-right looking behavior was scored off-line by a trained scorer, and 25% of trials were scored again by the same scorer and another experienced scorer blind to side of presentation of the target (see Experiment 1 for coder agreement). As in Experiment 1, we separately calculated the proportion of target looking for the pre- and post-naming phase of the experiment. Shapiro-Wilk tests confirmed that PTL did not depart from a normal distribution in both test phases (*p*s > .05).

### Results

Figure 2 shows the difference in the proportion of target looking before and after naming in the test block of this experiment. A 2 (Trial Phase) x 2 (Test Block) x 2 (Habituation Group) repeated measures ANOVA with Trial Phase (pre- vs. post-naming) and Test Block (pre- vs. post-training) as within-subjects factors and Habituation Group (Habituators (*N* = 13) vs. Non-Habituators (*N* = 17) as a between-subjects factor was run.

There was no main effect of Trial Phase, *F*(1, 28) = 0.06, *p* = .80 or Habituation Group, *F*(1, 28) = 0.013, *p* = .90, but there was a significant effect of Test Block, *F*(1, 28) = 0.11, *p* = .03, *η^2^* = .154, and no significant interactions between any of the factors tested (*p*s > .35). Planned post-hoc comparisons found no differences between the pre- and post-naming trial phases in the pre-test block, *t*(29) = − .81, *p* = .43, 95% CI [−0.12, 0.05], or in the post-test block, *t*(29) = 0.48, *p* = .64, 95% CI [−0.06, 0.11], suggesting both no prior knowledge of the word-object associations and a failure to learn these during the task.

Figure 3 shows the proportion of target looking over the course of the trial in the post-training test phase and highlights the absence of a systematic preference for target fixations following presentation of the label in this experiment.

### Discussion

Experiment 2 demonstrated that 10-month-old infants did not learn word-object associations when presented with multiple exemplars of an object category. It is possible that infants did not learn words because they found it difficult to generalize across multiple exemplars. However, the infant categorization literature demonstrates that infants at this age do learn categories in the presence of labels (e.g., Althaus & Westermann, 2016; Booth & Waxman, 2002; Plunkett, Hu & Cohen, 2008). Thus, infants’ failure to learn word-category associations in Experiment 2 is unlikely to stem from an inability to categorize. Rather, the lack of retention of these associations may be due to the increased cognitive load of encoding a category plus a label in Experiment 2 versus an individual object and a label in Experiment 1. Notably, this pattern of findings – retention after the same exemplar repeatedly but failure to retain after multiple exemplars – is the opposite of Twomey et al. (2014) findings for toddlers. We return to this issue in the General Discussion. Critically, forming and retaining word-object mappings is only part of the word learning process. Generalization of words to new category exemplars is also key. However, label generalization upon learning an object-label association in these naïve word learners has yet to be demonstrated. Thus, in Experiment 3, we provide infants with the challenging task of learning and then generalizing label-object mappings.

## Experiment 3: can 10-month-olds generalize newly-learned word-object associations to a novel category exemplar?

When infants learn a new word-object association, they must not only be able to recognize and associate familiar instances of the object with its label, but also to generalize this label to other unfamiliar instances of the object. With the caveats noted above, Junge et al. (2012) suggest that even very young infants may be able to generalize newly-learned labels to novel exemplars of the referent category: infants showed reduced brain potentials to novel or familiar instances of a trained object category accompanied by the label for these objects, while showing increased negativity when either the novel or familiar instance of the object category was presented with the label for the other object. Thus, Experiment 3 tests whether infants can extend newly-learned labels to a new exemplar. Experiments 1 and 3 were identical with regard to the training phases of the experiment (pre-test, interactive play phase, on-screen training phase), with the only difference being the final test phase, in which infants were presented with novel instances of the object category in Experiment 3.

### Method

#### Participants

30 infants (15 girls) with mean age of 9.97 months (range: 9.23– 10.67, *SD*: 0.30) were included in the final sample. Eight additional infants were tested but excluded due to fussiness. All participants were healthy, full-term infants, without any known visual or hearing problems, growing up in monolingual English-speaking households, recruited in the same way as the infants in Experiment 1. Participants were given a t-shirt or bib as a gift for their participation.

#### Stimuli

The same two word-object pairings were used in this study (*cow* and *horse*). During the pre-test phase, the interactive play phase and the on-screen training phase, infants were presented with exactly the same stimuli as the infants in Experiment 1. The only difference was the stimuli presented to infants in the test phase, in which infants were now presented with novel instances of the two object categories. These novel exemplars were indicated by adults as typical exemplars of the corresponding categories in our sets of cows (C2 – mean rating = 6.25/7) and horses (H2 – mean rating = 6.5/7). The results of the visual similarity study demonstrated that participants rated the novel cow exemplar as similar to the cow exemplar presented at training (mean rating = 5.83/7; *t*(19) = 8.42; *p* < .001). Similarly, participants rated the novel horse exemplar as similar to the trained horse exemplar (mean rating = 4.85/7; *t*(19) = 2.82; *p* < .05). As in Experiment 1, participants did not rate the visual similarity of the novel cow and novel horse exemplar highly (2.39/7; *t*(19) = − 3.8; *p* < .01). Furthermore, participants did not find the novel cow exemplar visually similar to the horse exemplar presented at training (3.35/7; *t*(19) = 4.22; *p* < .001), nor did they find the novel horse exemplar visually similar to the cow exemplar presented at training (2.9/7; *t*(19) = − 2.04; *p* = .05). Thus, the novel exemplars chosen as test items differed from one another to a similar extent as the exemplars presented at training (and in Experiment 1). The exemplars presented at test were rated to be more similar to the within-category exemplars presented during training than the across-category exemplars (presented during training or test).

As in Experiment 1, test items were stationary pictures of the objects against a white background. Again, all items were presented looking in two directions, by flipping the canvas horizontally for both still and moving images. Audio stimuli were identical to those in Experiment 1.

### Procedure

The experiment was divided into a pre-test phase, interactive play-phase, an on-screen training phase and a test phase. Aside from the test phase, all other phases of the experiment were identical to Experiment 1. Seventeen infants reached the habituation criterion. On average, infants in this experiment needed 4.37 sub-blocks (range: 3–5; *SD*: 0.85) to reach the habituation criterion.

#### Test phase

Infants were tested in a post-training test block identical to Experiment 1. The only difference to Experiment 1 was that infants were now presented with a novel instance of each object category at test.

#### Measures

Left-right looking was scored off-line by a trained scorer, and 25% of trials were scored again by the same scorer and another experienced scorer blind to side of target presentation (see Experiment 1 for coder agreement). As in Experiment 1, we separately calculated the proportion of target looking for the pre- and post-naming phase of the experiment. Shapiro-Wilk tests confirmed that PTL did not depart from a normal distribution in both test phases (*p*s > .05).

### Results

A 2 (Trial Phase) x 2 (Test Block) x 2 (Habituation Group) repeated measures ANOVA with Trial Phase (pre- vs. post-naming) and Test Block (pre- vs. post-training) as within-subjects factors and Habituation Group (Habituators (*N *= 17) vs. Non-Habituators (*N* = 13)) as a between-subjects factor was run. There were no main effects of Trial Phase, *F*(1,28) = 0.74, *p* = .40, or Test Block, *F*(1,28) = 0.11, *p* = .74, or Habituation Group, *F*(1,28) = 0.17, *p* = .68, or significant interactions between Trial Phase and Test Block, *F*(1,28) = 0.42, *p* = .52. No other interactions were significant. Planned post-hoc comparisons examined infants’ performance across the two test blocks. No differences between the pre- and post-naming trial phases were found in the pre-test block, *t*(29) = .14, *p* = .89, 95% CI [−.06, 0.07], or in the post-test block, *t*(29) = 0.80, *p* = .43, 95% CI [−0.04, 0.11], suggesting that infants did not know the words prior to training, nor did they extend the words to novel instances of the object category post-training (see Figure 2).

Figure 3 shows the proportion of target looking over the course of the trial in the post training test phase. The plot suggests chance level looking for the first part of the post-naming trial phase and a drop in target preference in the second part, which is typically interpreted as failure to recognize the word-object associations presented.

### Discussion

Experiment 3 investigated whether infants could generalize newly-learned associations to new category exemplars. Despite being exposed to identical training phases as in Experiment 1 above, infants in Experiment 3 showed no recognition of the association between the newly-learned label and the novel object instance. One account of the results so far is that 10-month-old infants may only acquire highly specific knowledge about new word-object associations: while they recognize a referent if they have previously encountered that exact item alongside a label, they fail to generalize that label to new exemplars. Equally, however, it is possible that our task made generalization difficult for infants, since we trained infants with items from two animal categories, i.e. cows and horses, which made them highly similar to each other. Thus, in Experiment 4, we made the task easier to maximize infants’ chances of generalization of a newly learned label to a novel exemplar of the referent category.

## Experiment 4: can 10-month-olds generalize newly-learned label-object association to a novel category exemplar when the two objects are highly perceptually dissimilar?

In Experiment 3, the degree of perceptual and conceptual overlap between cow and horse categories may have made it difficult for infants to discriminate between the two. Indeed, Bergelson and Aslin (2017) studied semantic representations in infants from 12 to 20 months of age and showed increasingly more detailed representations in the second year of life, but quite imprecise representations at the end of the first year. Infants at one year of age would look at a familiar object similarly when they heard it labeled and when they heard a related label (e.g. *foot* and *sock*), but during the second year of life children increasingly reduced their looking to related objects relying more on semantic fit. Additionally, Arias-Trejo and Plunkett (2010) showed that toddlers between 18 and 24 months struggled to show their knowledge of word-object associations when the target and distracter belong to the same global category (e.g. animals) and were perceptually similar. However, the same toddlers could identify word-object pairs when target and distracter come from different global categories, regardless of whether they were perceptually similar or not. In order to investigate whether infants’ failure to generalize in Experiment 3 was due to the perceptual and conceptual similarity between our cow and horse stimuli, Experiment 4 repeated Experiment 3, but with items from different global categories, specifically, animals and vehicles.

### Method

#### Participants

30 infants participated in the study (11 girls) with mean age of 10.00 months (range: 8.83– 11.07; *SD*: 0.51). All participants were healthy, full-term infants, without any known visual or hearing problems, growing up in monolingual English-speaking households. Nine additional infants were tested but not included in the final sample (6 due to equipment failure and 3 due to fussiness). Infants’ parents were recruited from a database of parents who had previously expressed willingness to participate. Parents’ travel expenses were reimbursed, and infants were given a storybook as a gift for their participation.

#### Stimuli

The stimuli for Experiment 4 were identical to the stimuli for Experiment 2 aside from an additional item, an airplane. Infants heard the shortened form, plane, in order for its word form to be similar in complexity to the other two, monosyllabic words, cow and horse. Airplane is also reported in the Wordbank database to be understood by only 6% of American infants at 10 months of age, which is similar to the other items used. Unlike Experiment 2, infants in Experiment 4 were presented with an animal and a vehicle; either a horse and an airplane or a cow and an airplane during training and test. Half of the infants were presented with a horse-airplane pair and half with a cow-airplane pair.

As with the cow and horse, a full set of airplanes was rated by adult native speakers of British English for their typicality and visual similarity to the cows and horses. From those airplanes, the two most typical exemplars were selected for the visual similarity analyses and infant testing, numbers 1 and 2 as presented in Table A1 of the Appendix; the first was presented during pre-test and training, and the second was presented at test. The two airplanes used in this experiment were rated as visually similar to one another (*M*: 4.25, *SD*: 2.30), as were the two horses (*M*: 4.85, *SD*: 1.35) and the two cows used (*M*: 5.40, *SD*: 1.60). Additionally, in order to compare the visual similarity of the specific between-category pairs (cow-horse, cow-airplane and horse-airplane), paired samples *t*-tests were run comparing each category pair to the other two. No differences were found between them, cow-horse: *t*(19) = 1.81, *p* = .09, cow-airplane: *t*(19) = − 1.98, *p* = .06, horse-airplane: *t*(19) = − 1.11, *p* = .28. Note that we did not ask participants to rate the visual similarity of items in this experiment across category boundaries given that the items stem from distinct global categories that were perceptually highly dissimilar to one another, for example cows and planes. The purpose of the visual similarity test was to ensure that the tokens presented at test were similar to the tokens presented at training in each of the categories tested, and equally similar in all the categories used.

#### Procedure

The procedure was identical to Experiment 3 with one item from each category presented during pretest and training, and a different typical item from each category presented at the post-training test phase. The only difference was the items used; instead of a cow and horse, either a cow-airplane pair or a horse-airplane pair was presented. Twenty-five infants reached the habituation criterion in this experiment. On average infants needed 3.30 sub-blocks (range: 2–5; *SD*: 1.02) to reach the habituation criterion.

#### Measures

Infant looking behavior was scored off-line by a trained scorer, and 25% of trials were scored again by another experienced scorer blind to side of target presentation

(see Experiment 1 for coder agreement). As in the other experiments, we separately calculated the proportion of target looking for the pre- and post-naming phase of the experiment. Shapiro-Wilk tests confirmed that PTL did not depart from a normal distribution in both test phases (*p*s > .05).

### Results

Figure 2 shows the difference in the proportion of target looking before and after naming in the pre-test and test blocks of this experiment. A 2 (Trial Phase) x 2 (Test Block) x 2 (Habituation Group) repeated measures ANOVA with Trial Phase (pre- vs. post-naming) and Test Block (pre- vs. post-training) as within-subjects factors and Habituation group (Habituators (*N* = 25) vs. Non-Habituators (*N *= 5)) as a between-subjects factor was run.

There were no main effects of Trial Phase, *F*(1,28) = 0.61, *p* = .44, or Habituation Group, *F*(1, 28) = 0.23, *p* = .64, and Test Block, *F*(1,28) = 0.64, *p* = .43, and no significant interaction between Trial Phase and Test Block, *F*(1,28) = 0.44, *p* = .51. No interactions were found with Habituation group. Data were collapsed across Habituation Group in subsequent analyses. Planned post-hoc comparisons found no differences between the pre- and post-naming phases in the pre-test block, *t*(29) = 0.06, *p* = .95, 95% CI [−0.09, 0.10], or in the post-test block, *t*(29) = − 1.35, *p* = .19, 95% CI [−0.15, 0.03], suggesting both an absence of prior knowledge of the labels and a failure to generalize labels to new category members in this brief experimental session (cow: *M *= 0.04, *SD *= 0.42; horse: *M *= 0.05, *SD *= 0.43; plane: *M *= 0.05, *SD *= 0.34).

#### Exploratory analysis

**Figure 4.**
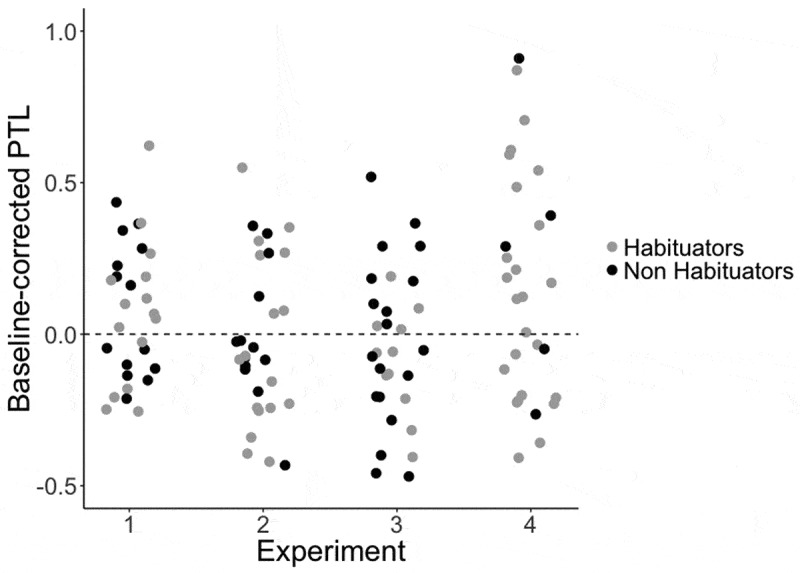
Target looking in Experiments 1–4 in the exploratory analysis: proportion of target looking in the second half of the post-naming window of the trial for test blocks in Experiments 1–4.

Figure 3 shows the proportion of target looking over the course of the entire trial in the post-training test phase, with no similar increase in target looking in the pre-test block. This peak in target looking toward the end of the post-naming phase of the trial could indicate a delayed target preference, similar to that found by Mather and Plunkett (2010) in 10-month-old infants. Indeed, given that children must generalize a novel label to an unfamiliar member of a newly learned category, it is likely that such responding is delayed relative to the faster processing shown in Experiment 1, where all infants need to do is match a familiar label to a familiar member of the category. Thus, we conducted a further exploratory analysis to establish whether this later peak in looking could correspond to a delayed label response. First, we divided the post-naming phase into two windows, one early window beginning 360 ms into the trial and continuing until 1070 ms after the onset of this window, and a second later window of equal duration (1070 ms) beginning at the end of the first window (3930 ms into the trial) and continuing until the end of the trial (5000 ms). We then examined whether there was a significant increase in preference for the target object from the pre-naming window (360 ms to 2500 ms into the trial) to the first or second post-naming window. While there was no significant increase in either the pre-test block or the first window of the post-test block (all *p*s > .610), we found a significant increase in target looking from the pre-naming window to the second time window in the post-test block, *t*(28) = − 2.24, *p* = .034, 95% CI [−0.29, −0.012]. Figure 4 shows the difference in target looking between the pre-naming and the second post-naming window.

### Discussion

Experiment 4 examined whether infants could generalize newly-learned labels to new exemplars of a given category when the two categories belonged to different global categories. Experiment 3 similarly tested generalization of newly-learned labels, but the visual stimuli (cows and horses) were perceptually similar and came from the same global category, and infants failed to show generalization. We hypothesized that if we increased the perceptual distance between the two to-be-learned categories, it might allow infants to exhibit their generalization abilities. In the main analysis, as in Experiment 2, infants did not show any increase in target fixations from pre-naming to post-naming in the test phase. However, while there was no effect of naming across the post-naming window as a whole, in an exploratory analysis, we divided the post-naming phase into two windows of equal duration and revealed a significant increase in target looking from the pre-naming phase to the second post-naming window. This increase may index a delayed label response which, if robust, suggests that 10-month-old infants can indeed learn two novel word-object associations and generalize these associations to a novel member of the newly-learned object category when the perceptual similarity of the two object categories to be learned is reduced. Clearly, the current finding is exploratory and should be interpreted cautiously; nonetheless Experiment 4 makes the intriguing prediction for future work that the ability to generalize labels immediate after learning a word-object association might be present as early as 10 months. In the next section, we discuss the implications of the results of these experiments taken together and situate these findings in the broader context of the literature on infant word learning.

### Cross-experiment comparison

We systematically compared the naming effect across the four experiments, separately analyzing the data aggregated across the entire post-naming phase and the data from the second half of the post-naming phases as conducted in Experiment 4.

#### Entire post-naming phase

We found no significant difference in the naming effect found in Experiment 1 and 2, *t*(57) = 1.86, *p* = .07, 95% CI [−0.01,0.23], and a significant difference between Experiment 1 and Experiment 3, *t*(57) = 2.19, *p* = .032, 95% CI [0.01, 0.23] There were no other significant differences between the naming effects found in the four experiments in this analysis (*p*s > .13).

#### Second half of the post-naming phase

We found a significant difference in the naming effect between Experiment 1 and Experiment 2, *t*(57) = 2.02, *p* = .049, 95% CI [0.01, 0.25, and between Experiment 1 and Experiment 3, *t*(57) = 2.19, *p* = .02, 95% CI = [0.02, 0.27], a significant difference between Experiment 2 and Experiment 4, *t*(57) = − 2.06, *p* = .04, 95% CI = [−.33, −.01] and a significant difference between Experiment 3 and Experiment 4, *t*(57) = − 2.42, *p* = .02, 95% CI = [−.36, −0.03]. There were no other significant differences between the naming effects found in the four experiments in this analysis (*ps* >.35; Figure 4).

These findings demonstrate that dividing the post-naming phase into two different windows may provide further insights into the temporal unfolding of infants’ looking behavior during the seconds following the presentation of the target word. In the analysis of the second window of the post-naming phase, significant differences were found between Experiment 1 and Experiments 2 and 3, as well as Experiment 4 and Experiments 2 and 3. The naming effect found in Experiment 1 is still evident in this exploratory analysis, but there is also evidence for recognition of target word-object associations in Experiment 4 which manifests itself in the form of a delayed response which otherwise would not have been evident. These exploratory results therefore point to an important avenue for future testing; specifically, infants at this young age may indeed respond to newly-learned category labels, but these responses may be delayed due to increased task demands.

## General discussion

Although research to-date suggests that young children are proficient word learners (e.g., Childers & Tomasello, 2002; Horst, Scott & Pollard, 2010; Houston-Price, Plunkett & Harris, 2005), the extent to which infants at the very beginnings of word learning can rapidly learn word-object associations remains unclear. The current study therefore set out to examine the limits of 10-month-old infants’ word learning. Experiment 1 found that infants could learn two novel word-object associations following minimal exposure to these associations in a brief experimental session. However, Experiments 2 and 3 revealed considerable constraints on infants’ word learning: infants did not map the word to an object category following training with multiple exemplars of the same object category (Experiment 2), and neither did they generalize the newly-learned labels to new exemplars of the object category (Experiment 3). Nevertheless, Experiment 4 found weak evidence that these infants could generalize a newly-learned label to a new member of the same category, as long as the two simultaneously introduced word-object associations involved perceptually dissimilar objects from distinct global categories, and infants were given adequate time to display their response.

### Early success in word learning and generalization

The findings across the four experiments extend our current understanding of early language development. First, building on previous studies with infants that have typically tested single word-object associations (e.g., Pruden et al., 2006), Experiment 1 showed that 10-month-old infants could simultaneously learn two novel word-object associations. Importantly, this experiment provides a stringent test of word learning at this early age. As argued above, testing learning of a single word-object association does not adequately distinguish between infants’ learning of distinct labels for distinct objects as opposed to learning that an object has a label. Furthermore, in this study, we ensured that infants were not familiar with the to-be-learned label-object associations prior to training, thereby allowing us to claim that infants learned the novel word-object association during the course of the experiment. However, it is likely that infants had seen cows or heard *cow* before being trained with our images and objects. Thus, we do not claim here that infants learned to map novel words to novel objects; that is, infants at the end of their first year are probably familiar with both the object categories and the sound forms used in these studies. Rather, in the context of our pre-test sessions, which showed that infants did not systematically respond to the word-object associations before training, we argue that infants learned a novel *association* between a word and its referent. It is possible, however, that infants’ prior familiarity with either the object or the label may have influenced the ease with which they learned the two word-object associations in the current study (Fennell, 2011; Kucker & Samuelson, 2012). Indeed, existing studies with novel objects and labels show that 10-month-old infants can learn novel word-object associations after substantially more training (Twomey & Westermann, 2018), or demonstrate rapid word-learning in older infants of 14 and 15 months of age (Schafer & Plunkett, 1998; Werker et al., 1998). Whether infants are similarly able to rapidly learn two completely novel word-object associations when the objects and the labels are completely unfamiliar remains an interesting challenge for future work.

Building on the findings of Experiment 1, Experiment 4 provided some evidence that infants could generalize newly-learned associations to new referents when test items were perceptually dissimilar and belonged to distinct global categories. While previous studies have examined whether infants can generalize labels to novel exemplars of familiar categories (Junge et al., 2012; Schafer, 2005), these studies have tested purely familiar word recognition (i.e., tested with familiar word-object associations) without examining infants’ knowledge of the word-object associations prior to training. The latter approach makes it difficult to examine the extent to which infants’ response at test is a result of the training provided during the experiment as opposed to their knowledge of these word-object associations prior to such training. In our study, however, we ensured that infants did not show recognition of the word-object associations prior to training and can, therefore, make stronger claims regarding infants’ learning of these associations during training. Under such conditions, we find that children can learn novel word-object associations and can, with some difficulty, generalize these learned associations to novel category exemplars, as long as the objects belong to different global categories (see Arias-Trejo & Plunkett, 2010, for similar results in toddler word recognition).

However, there are caveats to the findings in Experiment 4 that highlight constraints on infants’ word learning abilities at this young age. First, we note that infants in Experiment 4 did not show as consistent and robust a naming effect as infants in Experiment 1. Indeed, comparison of the time-course plots of responding in the post-test blocks in Experiments 1 and 4 highlights that infants in Experiment 1 displayed an early but consistent increase in looks to the target from around 300 ms post-onset of the target label for more than a second afterward. In contrast, in Experiment 4 infants showed a delayed spike in target preference toward the end of the trial, from around 1.5 seconds after the onset of the target label. This spike is reminiscent of the delayed responding found in infants at around the same age in experiments examining attention to novel objects upon hearing novel labels (Mather & Plunkett, 2010). Thus, Experiment 4 suggests that infants’ responses were potentially delayed by increased demands of encoding and mapping a label to a category rather than a single item as in Experiment 1.

Second, Experiment 2 further illustrates the limitations on infants’ word learning skills. When provided with richer perceptual input via multiple exemplars during training in Experiment 2, infants failed to learn labels. This finding diverges from the effect of variability on toddlers’ word learning (Twomey et al., 2014), and from tasks which have shown the benefit of multiple exemplars in other domains (e.g., Kovack-Lesh & Oakes. 2007; Quinn & Bhatt, 2010; Rost & McMurray, 2009). In the latter case, exposure to multiple exemplars supports categorization by allowing infants to compare the similarities and differences across category members to extract the properties critical to category membership (Oakes, Kovack-Lesh & Horst, 2009). In the case of word learning, however, exposure to multiple exemplars may place additional cognitive demands on the young learner, perhaps due to the young infants not being able to easily extract similarities across category members while nevertheless encoding all members with the same label. Thus, young infants may form separate links between each exemplar and the label, without as yet categorizing all exemplars under the label. In contrast, the older children in Twomey et al.’s (2014) study may more readily assign labels to categories, due to accumulated experience with label-category pairings, and therefore find it easier to make one-to-many label-object associations. Equally, it could be that while young infants can learn categories from visually variable stimuli (e.g., Quinn, Eimas, & Rosenkrantz, 1993), the increase in task demands associated with extra visual processing makes associating a label with that category – and retaining that association – more difficult for this young age group. More broadly, variability during learning may increase task difficulty for very young infants, while older children are able to capitalize on additional variability to support word learning.

Overall, our experiments suggest that word learning operates at multiple timescales: while infants can form and retain mappings in-the-moment (Experiments 1 and 4; see also Mather & Plunkett, 2010), linguistic and category information learned on a longer timescale is not yet sufficiently robust to support word learning when the learning environment changes (Experiments 2 and 3). Thus, the current study offers novel evidence supporting findings with older children which suggest that word learning begins as the formation of in-the-moment mappings between infants’ knowledge of words and the objects they refer to, and that these initially fragile mappings are reinforced over time until children display adult-like competence with a word (Carey & Bartlett, 1978; Kucker, McMurray, & Samuelson, 2015; McMurray, Horst, & Samuelson, 2012; Samuelson, Kucker & Spencer, 2016; Smith & Yu, 2008; Swingley, 2010; Yurovsky, Fricker, Yu, & Smith, 2014). This study highlights both the dynamic nature of word learning and the path that it must take from the association of a single label with a single object to the mapping of labels to object categories, illustrating the limits on infants’ early word learning skills.

## Appendix

**Table A1.** Mean typicality ratings (and standard deviations in parentheses) for the five most typical exemplars in the cow and horse categories, as well as the airplane category, as rated by adult native speakers of English.

Item	Cow		Horse		Plane	
1	6.71(0.61)		6.44(0.73)		7.00(0)	
2	6.25(0.77)		6.50(0.73)		6.80(0.41)	
3	5.93(1.16)		5.75(1.06)		6.20(1.57)	
4	6.44(0.81)		6.73(0.46)		5.44(2.39)	
5	5.31(1.82)		5.44(1.63)		5.12(2.63)

**Table A2.** Mean similarity ratings for the different pairs of cow stimuli presented in Experiment 2. Stimuli are depicted in Table A1 of the Appendix.

	C2	C3	C4	C5
C1	5.83	6.33	4.80	4.45
C2		4.75	6.28	5.63
C3			5.84	4.20
C4				5.17

**Table A3.** Mean similarity ratings for the different pairs of horse stimuli presented in Experiment 2. Stimuli are depicted in Table A1 of the Appendix.

	H2	H3	H4	H5
H1	4.85	6.28	6.00	4.30
H2		5.42	4.85	6.58
H3			6.11	5.05
H4				4.30

**Table A4.** Mean similarity ratings for the different pairs of cows and horse stimuli presented in Experiment 2. Stimuli are depicted in Table A1 of the Appendix.

	H1	H2	H3	H4	H5
C1	2.60	3.35	3.40	2.90	3.10
C2	3.15	2.39	3.75	2.90	2.85
C3	2.75	2.75	2.75	2.80	2.85
C4	2.55	2.55	3.15	2.95	2.75
C5	2.44	2.44	3.45	4.55	2.80
